# Metabarcoding Reveals Temporal Patterns of Community Composition and Realized Thermal Niches of *Thalassiosira* Spp. (Bacillariophyceae) from the Narragansett Bay Long-Term Plankton Time Series

**DOI:** 10.3390/biology9010019

**Published:** 2020-01-16

**Authors:** Tatiana A. Rynearson, Sarah A. Flickinger, Diana N. Fontaine

**Affiliations:** Graduate School of Oceanography, University of Rhode Island, South Ferry Road, Narragansett, RI 02882, USA; sflickinger89@gmail.com (S.A.F.); fontained@uri.edu (D.N.F.)

**Keywords:** community composition, diatom, diversity, high throughput sequencing, molecular ecology, phytoplankton, metabarcoding

## Abstract

Diatoms generate nearly half of marine primary production and are comprised of a diverse array of species that are often morphologically cryptic or difficult to identify using light microscopy. Here, species composition and realized thermal niches of species in the diatom genus *Thalassiosira* were examined at the site of the Narragansett Bay (NBay) Long-Term Plankton Time Series using a combination of light microscopy (LM), high-throughput sequencing (HTS) of the 18S rDNA V4 region and historical records. *Thalassiosira* species were identified over 6 years using a combination of LM and DNA sequences. Sixteen *Thalassiosira* taxa were identified using HTS: nine were newly identified in NBay. Several newly identified species have small cell diameters and are difficult to identify using LM. However, they appeared frequently and thus may play a significant ecological role in NBay, particularly since their realized niches suggest they are eurythermal and able to tolerate the >25 °C temperature range of NBay. Four distinct species assemblages that grouped by season were best explained by surface water temperature. When compared to historical records, we found that the cold-water species *Thalassiosira nordenskioeldii* has decreased in persistence over time, suggesting that increasing surface water temperature has influenced the ecology of phytoplankton in NBay.

## 1. Introduction

The combined activities of individual diatom species generate 40%–45% of oceanic primary production and influence the global cycling of silica and carbon [1,2,3]. Up to 200,000 diatom species are thought to exist [4,5], including species with different physiological and ecological characteristics including metabolic capability [6] and biogeographic range [5]. One of the challenges in accurately identifying the activities of individual species and their subsequent ecological and biogeochemical impacts is that many species are morphologically similar, or even identical, at the level of light microscopy [7,8].

Morphologically identical or "cryptic" species have been identified in the ecologically important diatom genus *Thalassiosira* [9,10]. Globally, *Thalassiosira* is one of the most abundant and diverse diatom genera [11] and includes species that are ecologically important components of phytoplankton communities in estuarine, coastal, and open ocean regions [12,13,14,15,16,17]. The identification of *Thalassiosira* species is known to be difficult, especially using light microscopy (LM) due to subtle differences in frustule morphology [18] and some *Thalassiosira* species are unidentifiable with LM methods [19]. While identification of *Thalassiosira* at the species level is possible using scanning electron microscopy (SEM) [13], this approach is generally not feasible for assessing large numbers of taxonomically complex field samples. Furthermore, small volume (e.g., 1 mL) counting methods typically used for LM identification of phytoplankton can underestimate species abundance by 20%–45% [20].

In many cases, community composition may be more accurately obtained through the use of molecular methods. For example, Hamsher et al. [10] were able to classify more *Thalassiosira* species from individual cells and colonies using molecular methods than LM, including rarely occurring species. Metabarcoding, or the high-throughput sequencing of PCR amplicons from an entire community, has been used to identify diatoms, including *Thalassiosira,* from complex communities [21,22,23]. At both the local and global scales, metabarcoding studies suggest that conventional LM methods have significantly underestimated diversity within the diatoms and they have revealed new and unidentified diatom ribotypes [21,24,25]. Generally, the focus of metabarcoding has been on either the entire phytoplankton community or on important functional groups, like diatoms, and most studies have reported taxonomy at the class, family and genus levels [22,25]. Few metabarcoding studies have focused on the ecological dynamics of individual species [21].

Here, we examined species richness and community composition in the diatom genus *Thalassiosira* using morphological count data and molecular data from the Narragansett Bay Long-Term Plankton Time Series (NBPTS) in Narragansett Bay (NBay), Rhode Island, USA [26,27]. The time series has collected weekly data on community composition since 1959. Historically, six *Thalassiosira* species were among the top 40 most abundant phytoplankton species in NBay [28]. *Thalassiosira nordenskioeldii,* the fifth most abundant phytoplankton species in NBay, was a characteristic member of the winter-spring bloom and *Thalassiosira rotula* was a characteristic fall blooming species [28]. With an understanding of the challenges involved in confidently identifying *Thalassiosira* to the species level, only a handful of taxa have regularly been recorded in the time series since 1959 (Table 1). This study used a metabarcoding approach combined with LM to investigate *Thalassiosira* community composition in NBay over a study period of 6 years and to address the following questions: (1) Which *Thalassiosira* species are present in NBay? (2) Are there temporal patterns in species composition and richness? (3) Do fluctuating environmental conditions drive observed temporal patterns? and (4) What are the realized thermal niches of species in NBay? To address these questions, we sequenced the 18S rDNA V4 region for 80 field samples collected over 6 years and compared this with species identification obtained using light microscopy. With this approach, *Thalassiosira* species not previously identified in NBay were identified and patterns of community composition were correlated with environmental conditions.

## 2. Materials and Methods

### 2.1. Field Sampling, DNA Extraction and Sequencing

As part of the NBPTS, weekly surface water samples were collected between December 2008 and December 2014 from the west passage of NBay (41°34.2′ N, 71°23.4′ W), a partially mixed estuary in the northwest Atlantic. Water samples were filtered in triplicate onto 0.22 μm pore size, 25 mm diameter ExpressPlus filters (MilliporeSigma, Burlington, MA, USA) and stored at −80 °C for later DNA extraction. Filter volume was dependent on the in situ Secchi depth; 100 mL of water were filtered per 1 m of Secchi depth which ranged from 1 to 6 m.

Previously extracted DNA from 68 monthly surface water samples collected between December 2008 and December 2014 was used here [21]. An additional 12 monthly samples collected between January and December 2014 were selected based on monthly maximum *Thalassiosira* cell abundance and DNA was extracted from those frozen filters following Canesi and Rynearson [21]. The final dataset contained 24 semi-monthly samples from 2014 and 80 field sampling dates overall (Table S1).

A 420 bp fragment within the variable V4 region of the 18S rDNA gene was amplified using primers D512 and D978rev [32] which amplify only diatoms and raphidophytes, enhancing our ability to amplify and detect rare diatom species. Primers were modified by the addition of Illumina specific adaptors: D512_illumina: 5’ TCGTCGGCAGCGTCAGATGTGTATAAGAGACAGATT CCAGCTCCAATAGCG 3’ and D978_illumina: 5’ GTCTCGTGGGCTCGGAGATGTGTATAA GAGACAGGACTACGATGGTATCTAATC 3’. The following reagents were used in 10 μL PCR reactions; 1X Bio-x-Act Short Mix (Bioline USA Inc., Taunton, MA, USA), 0.5 µM each forward and reverse primer, and approximately 0.3–2.7 ng DNA template. Reactions were amplified with a multi-step thermocycler protocol, consisting of a 2 min denaturing step at 94 °C, followed by 20 cycles of 30 s each at 94, 49, and 72 °C, followed by 15 cycles of 30 s each at 94, 67, and 72 °C, followed by 10 min at 72 °C. Technical variation of this PCR reaction was measured through triplicate amplification and sequencing of one arbitrarily selected sample (30 December 2014).

PCR amplicons were cleaned with Ampure XP beads (Beckman Coulter, Inc., Brea, CA, USA), quantified with the Qubit High Sensitivity DNA Assay Kit (Thermo Fisher Scientific, Inc., Waltham, MA, USA), amplified for an additional five cycles to add Nextera indices and adaptors (Illumina, Inc., San Diego, CA, USA) and cleaned again with Ampure XP beads. PCR products were pooled and quantified with the KAPA qPCR kit (Kapa Biosystems, Wilmington, MA, USA) prior to Illumina MiSeq sequencing with V2 chemistry (2 × 250 bp reads; Illumina, Inc., San Diego, CA, USA) at the University of Rhode Island Genomics and Sequencing Center.

### 2.2. Sequence Analysis

Paired end sequencing reads were first trimmed to remove low quality bases and Illumina adaptors using Trimmomatic PE [33], a 4 bp sliding window quality score of 20 and a minimum read length of 200 bp. Paired end sequences were merged using the BBmerge function from BBMap (Bushnell, Sourceforge.net/projects/bbmap/). Trimmed and merged sequences were filtered to a minimum length of 380 bp and a maximum expected error (based on phred quality scores) of 1 using USEARCH [34].

To identify taxa, filtered sequences were decomposed into minimum entropy decomposition (MED) nodes (equivalent to operational taxonomic units (OTUs)) [35], using a maximum entropy threshold of 0.0965 and a c value (maximum number of nucleotides with >0 entropy) of 4. Chimeric sequence nodes were identified and removed from results with UCHIME denovo [36] using an abundance skew value of 2.0. Taxonomy was assigned to remaining MED nodes using the BLAST method within the assign_taxonomy.py script from QIIME [37] and a custom in-house reference database of 38 *Thalassiosira* sequences curated from GenBank using only sequences from taxonomically verified specimens or submitted by taxonomists. Discarded MED nodes included those not assigned a taxonomy within the *Thalassiosira* reference database and those that contained fewer than 1% of total sequence reads per sample to control for sequencing error and to obtain meaningful ecological information.

A maximum likelihood tree of the custom reference database was constructed using the curated database and two outgroup species (*Skeletonema marinoi* and *Skeletonema menzelii*). Sequences were aligned using MUSCLE [38]. The TrN + G model [39] was selected based on Bayesian Information Criterion using jModelTest2 [40,41]. The maximum likelihood tree was created with PhyML V3.0 and 100 bootstrap replicates in Geneious [42,43].

All statistical analyses of high-throughput sequencing (HTS) reads used only presence-absence data because relative abundance of sequence reads does not correspond to relative abundance of species [21]. Variation within triplicate PCR reactions was assessed using ANOVA. The probability of a species to occur on any given month was calculated by summing the presence of that species for each month over the time series and normalizing by sampling effort. A Bray–Curtis similarity matrix of species occurrence probabilities was used to generate a multidimensional scaling plot (MDS) in PRIMER v6.1.6 (PRIMER-E Ltd., Albany, Auckland, New Zealand).

### 2.3. Comparison of HTS Data With LM Counts and Environmental Analyses

Light microscopy counts were obtained from the NBPTS [26] and the frequency and abundance of *Thalassiosira* species determined from December 2008 through December 2014 (Table S1). For seven dates across the 6-year dataset (23 May 2012, 25 June 2012, 2 July 2012, 1 August 2012, 5 September 2012, 5 October 2012, and 2 November 2012), light microscopy count data were unavailable. *Thalassiosira* species previously identified from NBay using LM were obtained from the modern (1999–present) [26] and historical time series (1959–1997) [27].

Species richness obtained using HTS was regressed against *Thalassiosira* abundance from the NBPTS using a least squares method and an ANOVA to test for significance of the slope. An ANOVA was used to test for significant differences in species richness with season (Winter: January–April, Spring: May–June, Summer: July–September, Fall: October–December). A multivariate trend correlation analysis (BIOENV) was used to correlate environmental factors with the Jaccard similarity index of community composition in PRIMER v6.1.6. Environmental data was obtained from the NBPTS (surface temperature, surface salinity, chlorophyll *a* [chl *a*], dissolved inorganic nitrogen [DIN], dissolved inorganic phosphorus [DIP], and silicate [Si]) [26]. For a small subset of samples (seven dates) for which chlorophyll *a* data was not available from the NBPTS, chl *a* data were obtained from the University of Rhode Island Marine Ecosystems Research Laboratory (MERL) Tank 98 bay sample dataset [44], which is collected weekly from the University of Rhode Island Graduate School of Oceanography dock (41.49°, −71.42°). This sample site is located 9.2 km south of Station II in the west passage of NBay and shares a similar physical environment with Station II. Additionally, temperature and salinity data for 15 September 2014 and nutrient data for 5 October 2014 were obtained from the MERL dataset [44]. Photosynthetically active radiation (PAR) data were obtained from the Narragansett Bay National Estuarine Research Reserve’s Narragansett Bay station (41.64°, −71.34°) [45]. Values for the seven environmental parameters were normalized in PRIMER v6.1.6 prior to analysis using BIOENV.

## 3. Results

### 3.1. Diversity and Occurrence of *Thalassiosira* Based on LM

**Figure 1:**
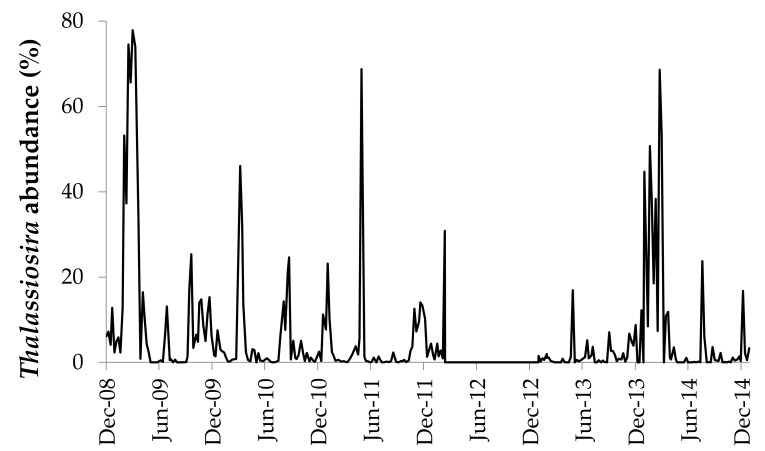
Percent abundance of *Thalassiosira* in weekly surface microplankton cell counts using light microscopy from the Narragansett Bay Long Term Plankton Time Series, December 2008–December 2014. Counts are not available from March–December 2012.

Based on LM records in the long-term dataset, *Thalassiosira* species were present in 85% of the weekly surface water samples collected between December 2008 and December 2014 (Figure 1). During that same time period, *Thalassiosira* cell abundance ranged from 0 to 1,683,000 cells L^−1^, with a median abundance of 13,000 cells L^−1^. The largest blooms of *Thalassiosira* most often occurred in February and March and constituted up to 78% of the total microplankton in NBay. Four *Thalassiosira* species or groups of species were recorded in the long-term data set during this time period; two were identified to the species level (*Thalassiosira nordenskioeldii*, *Thalassiosira punctigera*) and two represented multiple species (*Thalassiosira rotula*/ *Thalassiosira gravida* and *Thalassiosira* spp.). The last two categories represent species that are difficult or impossible to distinguish at the resolution of light microscopy.

### 3.2. Reference Database

**Figure 2:**
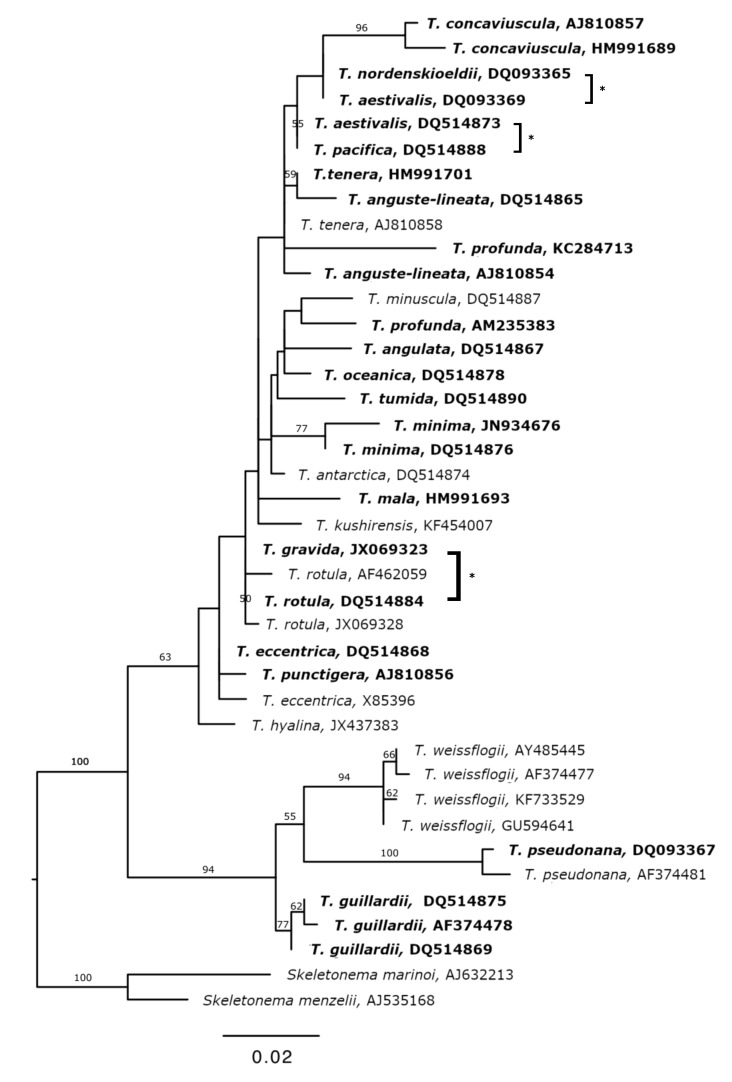
A maximum likelihood tree of *Thalassiosira* 18S V4 sequences included in the custom reference database. Bootstrap values (>50) are given at the nodes. Scale bar indicates genetic distance in substitutions per site. Bold labels denote sequences identified in the field samples analyzed here. Brackets with asterisks denote different species that are indistinguishable at the V4 region.

The 420 bp 18S rDNA V4 region did not clearly resolve all *Thalassiosira* species in the custom database (Figure 2). Five species were represented by multiple sequences with ambiguous placement on the tree, indicating that there are either species identification errors in GenBank or there is intraspecific variation at the V4 region for *Thalassiosira anguste-lineata*, *Thalassiosira profunda*, *Thalassiosira tenera*, *Thalassiosira eccentrica*, and *Thalassiosira aestivalis*. Three pairs of species could not be resolved at the 18S V4 locus because their sequences were not unique: *T. nordenskioeldii/T. aestivalis*, *Thalassiosira pacifica/T. aestivalis*, and *T. rotula/T. gravida.*

### 3.3. Sequencing Results

A total of 13.6 × 10^6^ read pairs from 82 samples (80 field samples and two amplification replicates) were sequenced (National Center for Biotechnology Information accession SRP078461). Reads per sample ranged from 58,448 to 281,234, with an average of 168,303 sequences per sample. An average of 13,016 sequences per sample (7.6%) survived the filtering and merging steps, with a range of 198 to 33,448. As the primers amplified all diatoms and raphidophytes, MED nodes that did not BLAST to our *Thalassiosira* reference database were discarded and not used in downstream analyses. Composition did not vary significantly among the three amplification triplicates (30 December 2014 field sample) (ANOVA, *p* = 0.74). Among replicates, there was no difference in the rank order of species (Table S2). *Thalassiosira minima* occurred in low abundance (1.1%) in only one of the triplicates.

All 80 field samples contained positive read matches to the genus *Thalassiosira* even when LM count data indicated that no *Thalassiosira* species were present. Sixteen *Thalassiosira* taxa were identified in the HTS data including nine that had not been identified using LM (Table 1). Six of those taxa have cell diameters under 15 µm, making them difficult to identify using LM at the 100× magnification used to obtain counts as part of the time series.

### 3.4. Seasonality of *Thalassiosira* Community Composition

**Figure 3:**
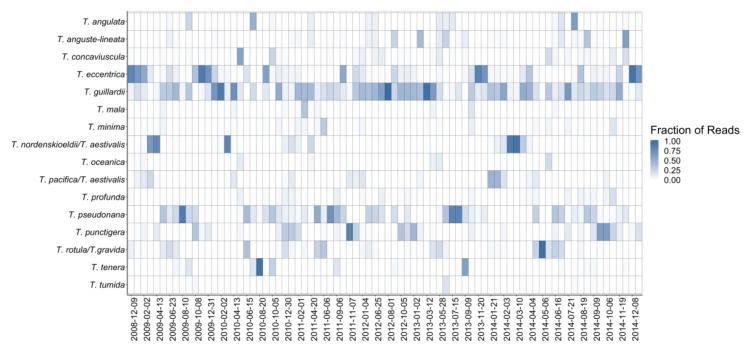
Heatmap showing relative abundance of sequence reads representing 16 *Thalassiosira* species identified using the metabarcoding approach. Samples were collected monthly between 2008 and 2013 and semi-monthly for 2014. Sample dates are represented in columns.

**Figure 4:**
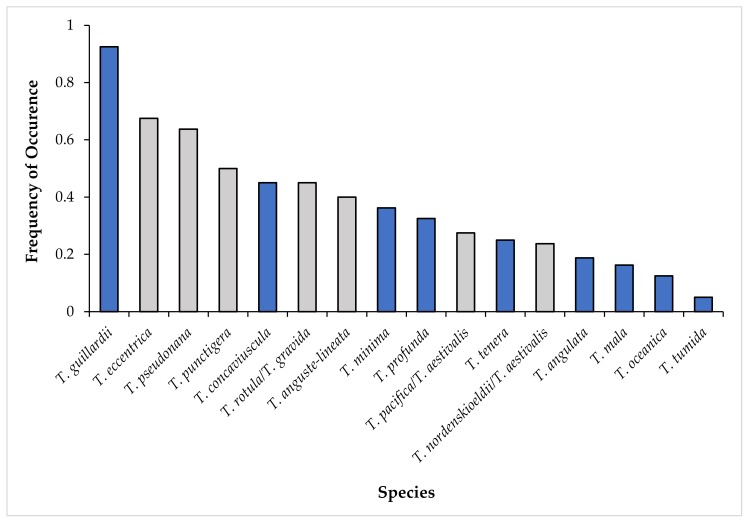
The frequency of occurrence of each species across all 80 sequenced field samples. Blue bars indicate newly identified species in Narragansett Bay.

The 16 *Thalassiosira* species observed in the HTS dataset had different patterns (Figure 3) and frequencies of occurrence (Figure 4). The majority of species did not occur frequently; only three species appeared in more than 50% of field samples (Figure 4). *Thalassiosira guillardii* was the most frequent, appearing in 93% of field samples. *T. eccentrica* was the second most frequent (68%). *Thalassiosira pseudonana* was present in 64% of samples and all other species appeared less frequently.

**Figure 5:**
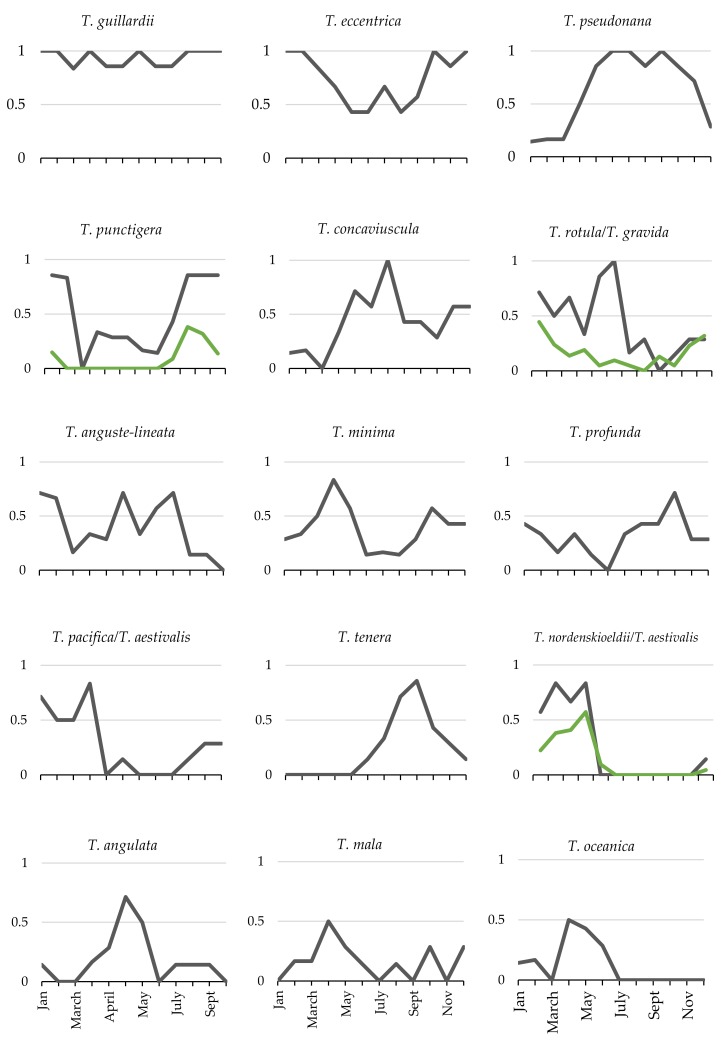
Monthly probability of occurrence of each species in the sequenced field samples listed in order of abundance (cf. Figure 4). Green lines represent probability of occurrence data for species identified using LM. The least frequently occurring species, *Thalassiosira tumida*, is not shown.

A variety of occurrence patterns was observed (Figure 5). The two most frequently occurring species, *T. guillardii* and *T. eccentrica*, had a high probability of occurrence throughout the year. In contrast, the third most frequently occurring species, *T. pseudonana*, had a strong seasonal signal with at least 85% occurrence during the spring and summer months (May–September). The remaining species generally had a seasonal signal of occurrence. One set of species had elevated probabilities of occurrence during the late fall and winter (*T. nordenskioeldii/T. aestivalis, T. pacifica/aestivalis*, and *Thalassiosira punctigera*). Another set of species occurred most frequently in late spring–summer (*T. rotula/T. gravida*, *Thalassiosira concaviuscula*, *Thalassiosira angulata*, and *Thalassiosira oceanica*). In mid-summer and fall, *T. tenera* and *Thalassiosira tumida* occurred more frequently whereas *Thalassiosira minima*, *Thalassiosira mala*, and *T. profunda* occurred more frequently in spring and fall.

Notably, two of the species identified using both HTS and LM followed similar seasonal patterns (*T. punctigera*, *T. nordenskioeldii*, Figure 5). High-throughput sequencing and LM for *T. rotula/gravida* also followed roughly similar seasonal patterns with the exception that HTS indicated the highest probability of occurrence in June, a time when LM data suggested low levels of occurrence.

**Figure 6:**
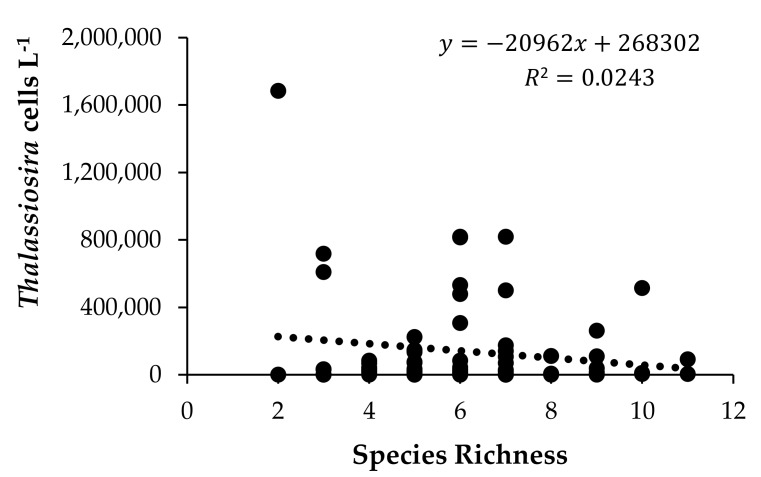
Species richness of *Thalassiosira* identified using HTS compared to cell counts obtained using LM for 80 field samples between 2008–2014.

Species richness varied from 2 to 11 and some species did not co-occur. For example, the winter *T. nordenskioeldii/T. aestivalis* group never co-occurred with the late summer *T. tenera* or *T. tumida.* There was no significant relationship between species richness and the abundance of *Thalassiosira* obtained from the NBPTS (*p* = 0.18) (Figure 6).

**Figure 7:**
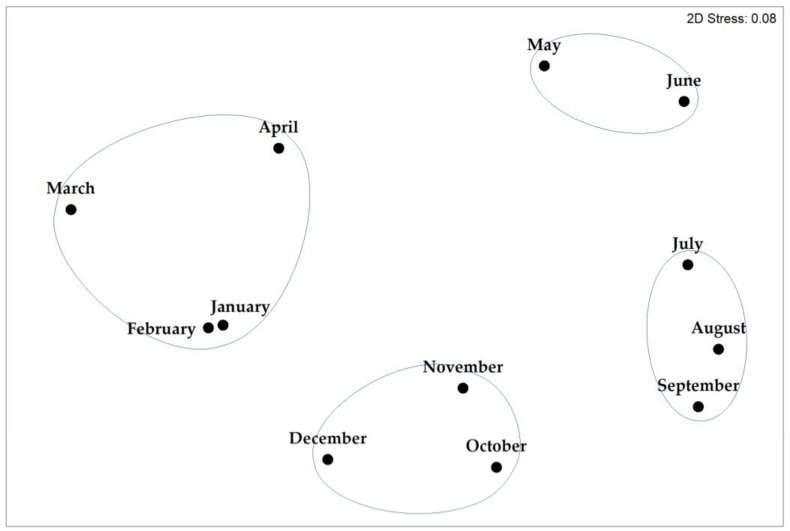
Multidimensional scaling plot (MDS) of Bray–Curtis similarity, based on the monthly probability of occurrence of each *Thalassiosira* species in the HTS dataset. Four seasonal groups are apparent at the 70% similarity level: winter (January–April), spring (May–June), summer (July–September), and fall (October–December).

To determine if there were similarities among species assemblages collected at different times of year, a Bray–Curtis similarity matrix of monthly species occurrence probabilities was calculated. A multidimensional scaling plot of the Bray–Curtis similarity matrix revealed four distinct seasonal assemblages: winter (January, February, March, April), spring (May, June), summer (July, August, September) and fall (October, November, December) (Figure 7). There was no significant difference in species richness among seasons (*p* = 0.63) suggesting that while species composition shifted throughout the year, richness did not.

### 3.5. Environmental Factors Associated With Species Occurrence

**Figure 8:**
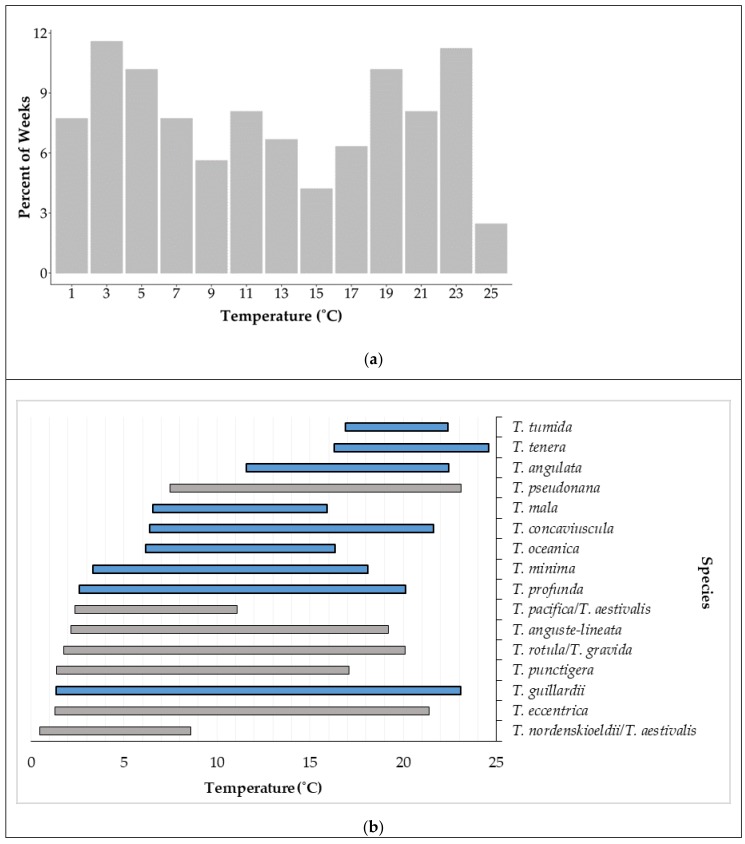
(**a**) Frequency distribution of surface water temperatures, in 2 °C bins, measured at the Narragansett Bay Long Term Plankton Time Series site, December 2008–December 2014. Value on *x*-axis represents the middle of each bin (i.e., 1 °C for the >0–≤2 °C bin). (**b**) Observed temperature ranges for the *Thalassiosira* species recovered from the HTS data. Blue bars indicate newly identified species in Narragansett Bay.

Environmental factors in NBay varied widely from December 2008 to December 2014 (Table 2, Figure S1). Among the variables examined, surface temperature explained 31.2% of the variation in *Thalassiosira* community composition (Table 3). The inclusion of additional variables decreased the correlation. Surface temperature in NBay from 2008 to 2014 showed a bimodal distribution with peaks between 2–4 and 22–24 °C which each occurred about 12% of the time (Figure 8a). The realized thermal niches of most species were broad, although a few were narrow. For example, the *T. nordenskioeldii/T. aestivalis* group only occurred between 0.5 and 8.5 °C (Figure 8b). *Thalassiosira mala* and *T. oceanica* were observed only at moderate temperatures (6.6–15.9 °C and 6.2–16.3 °C, respectively), while *T. tenera* and *T. tumida* were only observed at the warmest temperatures observed in NBay (16.3–24.6 °C).

## 4. Discussion

### 4.1. Utility of the 18S V4 Region for Species Identification in the Genus *Thalassiosira*

As few metabarcoding studies have focused on individual taxa and their ecological dynamics, we evaluated the utility of focused metabarcoding analysis of a single genus. By using diatom-specific primers that amplified the 18S rDNA V4 region [32], we identified *Thalassiosira* species more frequently using HTS than LM. *Thalassiosira* species were recovered from all 80 HTS samples but only from 70 samples using LM counts of 1 mL volumes. Notably, the metabarcoding data were obtained from amplified DNA extracted from 100–300 mL filtered seawater, thus capturing species present at far less than 1 cell mL^−1^.

The 18S V4 region amplified here does not resolve all *Thalassiosira* species, since some have identical sequences at this locus (e.g., *T. nordenskioeldii* and *T. aestivalis*, *T. pacifica* and *T. aestivalis*, *T. rotula* and *T. gravida*). In total, our curated reference database differentiated 33 of 38 *Thalassiosira* species. Additionally, for some species in the genus, the 18S sequences are not present in public databases. For example, *Thalassiosira decipiens* is recorded in the NBPTS [27] but could not be detected in the sequence data because the 18S V4 sequence is not available in GenBank. This limitation is present for all barcoding loci used to date, including the 18S rDNA and highlights the need for continued efforts to obtain sequence data from taxonomically verified species.

Another limitation of the HTS metabarcoding approach is variation in 18S copy number among species. 18S gene copy number can vary from 1 to 12,000 copies cell^−1^ in phytoplankton, and more specifically, can vary by an order of magnitude among *Thalassiosira* species [46]. For the large majority of *Thalassiosira* species, copy number per species is unknown. Although some have argued that abundances observed in LM and HTS efforts are comparable [25], experiments using mock communities revealed that relative abundance obtained using HTS differs significantly from in situ abundance [21,47]. Here, all statistical analyses and inferences were conducted using only presence-absence data to avoid the pitfalls associated with variable copy number.

### 4.2. Species Diversity of *Thalassiosira* in Narragansett Bay

The diversity of *Thalassiosira* species in NBay is greater than previously realized. Of the 16 species identified using HTS, nine had not been historically documented in the NBPTS using LM. A combination of both HTS and LM data from 1959 through 2014 yielded a total of 20 *Thalassiosira* species or species groups (e.g., *T. rotula/gravida*). This is comparable to *Thalassiosira* species diversity found in other regions. For example, taxonomic studies using different combinations of LM, scanning electron microscopy, and DNA sequencing identified 21 *Thalassiosira* species in the North Sea [13] and 18 *Thalassiosira* species in a Scottish sea loch [16]. This suggests that even with an incomplete reference database, it is possible that a majority of *Thalassiosira* species in NBay were identified using the 18S V4 region.

Several of the newly recorded species in NBay are small (<15 μm in diameter) (*T. guillardii*, *T. mala*, *T. minima*, *T. oceanica*, *T. profunda*, *T. pseudonana*; Table 1) which may explain why they were not identified using LM at 100×, the magnification used to obtain species composition and abundance for the NBPTS. In fact, at least one of these small species, *T. profunda*, is not identifiable with LM [19]. The presence of these small species in NBay is consistent with their known habitats, which includes estuaries [16,30,48].

Interestingly, the second most abundant *Thalassiosira* species recorded in the historic dataset (and sixth most abundant phytoplankton species) [28] was characterized as a small (≈10 μm in diameter), unidentified *Thalassiosira* species that appeared in NBay beginning in 1967 and became both frequent and abundant. The most frequent species in the HTS data was the small *Thalassiosira guillardii* (4–14 μm in diameter). It was present during all months of the year and its probability of occurrence in any given month ranged from 83% (March) to 100% (multiple months). In other temperate estuaries, this species can occur frequently and in high cell numbers [30,48]. Other small diameter species in the HTS dataset, *T. minima* and *T. profunda*, appeared in >33% of all samples and have been observed previously from coastal habitats [30,49]. Given the small size of these species and the difficulty in distinguishing among them using LM, it is possible that a combination of species was present in the historic dataset and contributed to the highly abundant *Thalassiosira* spp. category. Together, they represent a set of small but potentially ecologically important diatoms in NBay, particularly given recent work highlighting the important role of nanoplanktonic diatoms in spring blooms and carbon export across the global ocean [50].

One of the smallest species identified in the HTS dataset is *T. pseudonana* (2.3–5.5 μm diameter) [29]. Although it was not identified in NBPTS LM counts during this study period, it was the sixth most abundant *Thalassiosira* species recorded in the time series from 1959 to 1980 [28] and was present primarily in June and July [27]. In the HTS data, *T. pseudonana* was the third most frequently occurring species with highest occurrences between May and November (100% in June, July, and September), suggesting that this is primarily a warm water species in NBay, although its realized thermal niche spanned from 7.5 to 23 °C. *Thalassiosira pseudonana* can be a dominant spring taxon in Chesapeake Bay [51] and forms blooms in July and August in a brackish estuary off the Bay of Biscay [52]. This species is an important model organism with a fully sequenced genome [53]. Our results suggest that this species could be a more frequent and important contributor to estuarine phytoplankton communities than previously recognized.

A newly recorded, but larger, species in the dataset is *T. tumida.* Notably, this species has only been recorded from the Southern Ocean [29]. While this could suggest that the range of this species is greater than previously thought, samples with *T. tumida* were collected in the fall and summer when water temperatures were 16.9–22.4 °C. It is unlikely that the cause was either sequencing error since this taxon comprised 3%–15% of the sequence reads when it was present or misidentification in Genbank since multiple *T. tumida* accessions align and there are no other named species with a V4 region matching the *T. tumida* sequence exactly. A more likely scenario is that another, as yet unidentified *Thalassiosira* species shares the same 18S V4 sequence as *T. tumida* [9].

### 4.3. Seasonal Patterns of Species Composition

Seasonal patterns were apparent both for individual species and whole communities of *Thalassiosira*. Multidimensional scaling analysis of the HTS data revealed four assemblages that corresponded with season. Of the environmental parameters that were analyzed, temperature was most highly correlated with variations in community composition. This result is consistent with other studies in NBay that have identified temperature as a key correlate of species composition including within the genus *Skeletonema* [21] and across all phytoplankton from 1959 to 1980 [28]. Narragansett Bay is a temperate estuary sited between the cold Boreal waters of the Gulf of Maine to the North and warmer waters of the mid-Atlantic Bight to the South and it experiences great excursions in temperature annually, from 0.5 to 24.6 °C during this study period.

Realized thermal niches of phytoplankton can be difficult to obtain given that many species cannot be detected using LM if cells are small and/or rare. The 6-year time frame of this study, broad temperature range in NBay, and metabarcoding approach allowed us to examine realized thermal niches in *Thalassiosira*, particularly for those species that were newly identified in the bay. The small diameter, newly identified *T. guillardii* could be characterized as eurythermal because it was present in all samples across all water temperatures. Alternately, the broad temperature range may be a sign that the *T. guillardii* V4 region represents a closely-related species complex, similar to that found in the diatom genus *Skeletonema*, which is comprised of many species with identical V4 sequences and in NBay, could appear eurythermal using the V4 [21]. Other species had realized thermal niches that were more restricted. At the coldest temperatures (≤1 °C), only *T. nordenskioeldii* (see below) was present in the bay and at 6 °C, over half the *Thalassiosira* species observed in NBay were present. The warmest water temperatures had a similar pattern where only the newly identified *T. tenera* was present at temperatures above 23 °C but at water temperatures of 21 °C, about half of the species were present. Interestingly, surface waters were ≤6 °C 30% of the time and ≥20 °C 21% of the time, providing a substantial time frame for the proliferation of species that can survive at those temperatures. The range of realized thermal niches in NBay parallels observations by Thomas et al. [54] who found an exceptionally broad range of temperatures for optimal growth (≈6–30 °C) in phytoplankton species collected from latitudes similar to NBay (41° N).

*Thalassiosira nordenskioeldii* is a characteristic winter species in NBay [28]. Although *T. nordenskioeldii* could not be distinguished from *T. aestivalis* at the V4 region, the observed temperature range for the *T. nordenskioeldii/T. aestivalis* group was 0.5–8.5 °C, which is within the temperature range (0.5–15 °C) identified for *T. nordenskioeldii* in NBay [28] and the laboratory [55]. In contrast, *T. aestivalis* has not generally been identified in the NBPTS aside from a handful of observations (seven times from 1959–1997) and is recognized as a temperate to warm water species [29], suggesting that the taxon identified here is likely *T. nordenskioeldii*. In an analysis of the NBPTS samples collected from 1959 to 1980, *Thalassiosira nordenskioeldii* was identified as the most abundant *Thalassiosira* species in NBay and the fifth most abundant diatom species overall [28]. In the 2008–2014 samples analyzed here, *T. nordenskioeldii* was not present at all from 2012 to 2013 in both the HTS and LM datasets. This species was present in 32% of the LM counts between 1959 and 1980 [28] but only in 15% of the LM counts during this study period. Given that the 2008–2014 time period of this study would likely capture one of the ≈ 5 year cycles in abundance that *T. nordenskioeldii* undergoes in NBay [28], the recent LM and HTS datasets suggest a decrease in persistence and possible decrease in ecological importance of this cold water species in NBay over the last 55 years. This is consistent with the nearly 2 °C warming of wintertime waters in NBay over the same time frame [56].

Other environmental factors that could significantly influence the composition and seasonality of *Thalassiosira* species in NBay include large-scale climatic processes, grazing by zooplankton, and spore formation. Large-scale processes such as the meandering of the Gulf Stream and the North Atlantic Oscillation are known to impact the size and timing of NBay summer *Skeletonema* blooms [57] and may also affect *Thalassiosira* species. Zooplankton grazing also has the potential to significantly alter community composition. Microzooplankton grazers consume an average of 96% of primary production in NBay [58] and the ctenophore *Mnemiopsis leidyi* can exert significant control on the plankton community in NBay [59]. Finally, community composition and seasonality can be significantly influenced by spore formation, which occurs in some *Thalassiosira* species [60] (reviewed in McQuoid and Hobson 1996). *Thalassiosira guillardii* is not known to form resting stages which may explain its persistence in the water column. In contrast, other frequently occurring species in NBay do form resting stages, including *T. nordenskioeldii*, *T. rotula*, *T. pseudonana*, and *T. minima* [60,61]. Together with large fluctuations in water temperature, resting stages may play an important role in the seasonality of species in NBay.

## 5. Conclusions

Using a combination of LM and metabarcoding approaches to analyze 6 years of data from the NBPTS, we found that *Thalassiosira* species diversity in NBay is greater than previously recognized. Much of this newly identified diversity can be attributed to several smaller sized species that are very frequent, have been overlooked using light microscopy, and may play an important ecological role. Overall, *Thalassiosira* species composition was most highly correlated with temperature. Analysis of realized thermal niches suggests that at least four types of species occur in NBay including cold, warm, and intermediate water species, as well as eurythermal species that can tolerate nearly a 25 °C temperature range. Given that temperatures in NBay are warming at a rate of nearly 0.2 °C per decade [56], there may be an increase in species that prefer warmer temperatures, such as *T. tenera,* and a continuation of the decrease we observed in persistence and possible ecological role of Arctic/Boreal species such as *T. nordenskioeldii*.

## Figures and Tables

**Table 1:** Cell diameter and species range from Tomas [29], except where noted, for all *Thalassiosira* species identified with light microscopy (LM) (1959–1997 [27] and 1999–2014 [26]) and with high-throughput sequencing (HTS) (2008–2014). Species were either identified uniquely (x), identified as part of a group of indistinguishable species (*), not detected because sequence not available in reference database (-) or not detected (blank). Numbers indicating the frequency of observation (no. of weeks observed) are included for rarely occurring species (≤10 observations) in the light microscopy dataset.

Species	Cell Diameter (µm)	Geographic Range	LM 1959–1997	LM 1999–2014	HTS 2008–2014
*T. aestivalis*	14–56	Warm to temperate	7		*
*T. angulata*	12–39	Cold to temperate ^1^			x
*T. anguste-lineata*	14–78	Cosmopolitan		1	x
*T. concaviuscula*	14–56 ^1^	Neritic, cold to temperate ^1^			x
*T. decipiens*	9–40	Cold to temperate	x		-
*T. eccentrica*	15–110	Cosmopolitan, except polar zones	2	1	x
*T. gravida*	17–62	Cold to temperate	x	*	*
*T. guillardii*	4–14 ^2^	Marine and brackish ^2^			x
*T. hyalina*	16–45	Cold to temperate	1		
*T. mala*	4–10	Warm to temperate			x
*T. minima*	5–15	Cosmopolitan, except polar zones			x
*T. nordenskioeldii*	10–50	Cold to temperate	x	x	*
*T. oceanica*	3–12	Mainly warm waters			x
*T. pacifica*	7–46	Cosmopolitan, except polar zones	9		*
*T. profunda*	1.8–5 ^3^	Cosmopolitan ^2^			x
*T. pseudonana*	2.3–5.5	Cosmopolitan	x		x
*T. punctigera*	40–186	Warm to temperate	8	x	x
*T. rotula*	8–55	Cosmopolitan	x	*	*
*T. subtilis*	15–32	Warm to temperate	5		-
*T. tenera*	10–29	Cosmopolitan ^4^			x
*T. tumida*	21–137	Southern cold water region			x
*T. spp*			x	x	

^1^ M Hoppenrath et al. [13]; ^2^ GR Hasle [19]; ^3^ J Belcher and E Swale [30]; ^4^ GR Hasle and G Fryxell [31].

**Table 2:** Range and median of environmental variables across the 80 field samples (2008–2014).

Environmental Variable	Range	Median
Sea surface temperature (°C)	0.5–24.6	13.9
Sea surface salinity	18.60–32.39	29.93
Average daily PAR (mmol m^−2^)	16.21–572.05	247.87
Dissolved inorganic nitrogen (µM)	0.14–15.80	2.61
Dissolved inorganic phosphorus (µM)	0.04–1.87	0.72
Silicate (µM)	0.05–38.12	11.46
Chlorophyll a (μg L^−1^)	0.23–29.80	5.14

**Table 3:** Multivariate correlation of *Thalassiosira* species composition with environmental factors.

Correlation	Environmental Variables
0.312	Surface Temperature
0.309	Surface Temperature, DIP
0.298	Surface Temperature, Si
0.296	Surface Temperature, Surface Salinity

## Supplementary Materials

The following are available online at https://www.mdpi.com/2079-7737/9/1/19/s1, Table S1: List of field samples. Table S2: Species identified in sequencing triplicates. Figure S1: Monthly averages of environmental variables for the study period (2008–2014).
