# Supplementary material for: Metabarcoding Reveals Temporal Patterns of Community Composition and Realized Thermal Niches of Thalassiosira Spp. (Bacillariophyceae) from the Narragansett Bay Long-Term Plankton Time Series
Source: Biology (Basel). 2020 Jan 16;9(1):19. doi: 10.3390/biology9010019 (PMC7168904; doi:10.3390/biology9010019)
Supplement: Supplementary file 1 [file biology-09-00019-s001.zip › Rynearson_et_al_SUPPLEMENTARY DOCUMENTS.docx]

**SUPPLEMENTARY DOCUMENTS**

Supplementary Table 1: List of 80 field samples collected from Narragansett Bay from December 2008 through December 2014 including date of sample collection, total phytoplankton abundance and *Thalassiosira* genus abundance quantified by light microscopy. The list includes dates when samples for sequencing were collected but for which no phytoplankton abundance data is available (indicated by NA). Monthly samples were sequenced for 2008-2013, with the exceptions of July 2011 and February-April 2012, when samples were not collected. Semi-monthly samples were sequenced for 2014.

| Sample Collection Date | Total Phytoplankton Abundance (cells/liter) | *Thalassiosira* Abundance (cells/liter) |
| --- | --- | --- |
| 12/9/2008 | 1866000 | 135000 |
| 1/27/2009 | 242000 | 31000 |
| 2/2/2009 | 207000 | 110000 |
| 3/3/2009 | 921000 | 717000 |
| 4/13/2009 | 5147000 | 533000 |
| 5/27/2009 | 1153000 | 0 |
| 6/23/2009 | 4680000 | 306000 |
| 7/27/2009 | 10784000 | 70000 |
| 8/10/2009 | 25849000 | 0 |
| 9/4/2009 | 17383000 | 16000 |
| 10/8/2009 | 541000 | 35000 |
| 11/23/2009 | 438000 | 67000 |
| 12/31/2009 | 728000 | 22000 |
| 1/26/2010 | 19612000 | 34000 |
| 2/2/2010 | 10633000 | 23000 |
| 3/29/2010 | 6085000 | 140000 |
| 4/13/2010 | 49346000 | 112000 |
| 5/28/2010 | 4942000 | 13000 |
| 6/15/2010 | 12709000 | 84000 |
| 7/19/2010 | 18144000 | 75000 |
| 8/20/2010 | 8015000 | 1683000 |
| 9/15/2010 | 599000 | 6000 |
| 10/5/2010 | 2101000 | 107000 |
| 11/16/2010 | 1474000 | 6000 |
| 12/30/2010 | 1179000 | 91000 |
| 1/11/2011 | 4991000 | 514000 |
| 2/1/2011 | 3539000 | 14000 |
| 3/9/2011 | 2840000 | 2000 |
| 4/20/2011 | 14207667 | 262000 |
| 5/31/2011 | 4361000 | 2000 |
| 6/6/2011 | 19456000 | 29000 |
| 8/19/2011 | 1861000 | 43000 |
| 9/6/2011 | 3590000 | 0 |
| 10/3/2011 | 2460000 | 2000 |
| 11/7/2011 | 454000 | 33000 |
| 12/7/2011 | 165000 | 17000 |
| 1/4/2012 | 699000 | 11000 |
| 5/23/2012 | NA | NA |
| 6/25/2012 | NA | NA |
| 7/2/2012 | NA | NA |
| 8/1/2012 | NA | NA |
| 9/5/2012 | NA | NA |
| 10/5/2012 | NA | NA |
| 11/2/2012 | NA | NA |
| 1/2/2013 | 45200 | 700 |
| 2/12/2013 | 8272800 | 31000 |
| 3/12/2013 | 1157000 | 0 |
| 4/2/2013 | 807800 | 0 |
| 5/28/2013 | 478000 | 2500 |
| 6/19/2013 | 15745200 | 815000 |
| 7/15/2013 | 48604100 | 0 |
| 8/16/2013 | 2769200 | 4000 |
| 9/9/2013 | 5512100 | 147000 |
| 10/28/2013 | 199700 | 400 |
| 11/20/2013 | 851900 | 40000 |
| 12/2/2013 | 809000 | 71000 |
| 1/21/2014 | 987700 | 501000 |
| 1/27/2014 | 1239500 | 479000 |
| 2/3/2014 | 944000 | 175000 |
| 2/24/2014 | 889300 | 610000 |
| 3/10/2014 | 409500 | 0 |
| 3/31/2014 | 4297900 | 45000 |
| 4/4/2014 | 12428800 | 84000 |
| 4/21/2014 | 6082100 | 39000 |
| 5/6/2014 | 746000 | 0 |
| 5/27/2014 | 93800 | 1000 |
| 6/16/2014 | 6100267 | 0 |
| 6/23/2014 | 19872200 | 21000 |
| 7/21/2014 | 4286100 | 18000 |
| 7/28/2014 | 13656400 | 819000 |
| 8/19/2014 | 10642500 | 4000 |
| 8/25/2014 | 5256900 | 819000 |
| 9/9/2014 | 446000 | 4000 |
| 9/15/2014 | 900000 | 224000 |
| 10/6/2014 | 2469000 | 4000 |
| 10/27/2014 | 565000 | 0 |
| 11/19/2014 | 435000 | 3000 |
| 11/25/2014 | 288000 | 4000 |
| 12/8/2014 | 192100 | 32200 |
| 12/30/2014 | 609000 | 20000 |
